# Objectively-assessed physical activity and self-reported activity pacing in adults with multiple sclerosis: A pilot study

**DOI:** 10.1177/02692155211024135

**Published:** 2021-06-16

**Authors:** Ulric S Abonie, John Saxton, Katherine Baker, Florentina J Hettinga

**Affiliations:** 1 Department of Physiotherapy and Rehabilitation Sciences, University of Health and Allied Sciences, Ho, Volta Region, Ghana; 2 School of Sport, Rehabilitation and Exercise Science, University of Essex, Colchester, UK; 3 Department of Sport, Exercise and Rehabilitation, Northumbria University, Newcastle upon Tyne, UK

**Keywords:** Multiple sclerosis, accelerometer, energy modulation

## Abstract

**Objective:**

To examine the association between self-reported activity pacing (a strategy to manage fatigue symptoms) and objectively-measured physical activity behaviours in adults with multiple sclerosis.

**Design:**

Single cross-sectional study

**Setting:**

Multiple sclerosis rehabilitation centre in Colchester, United Kingdom.

**Subjects:**

Twenty-one adults (59 ± 9 years) with multiple sclerosis.

**Main measures:**

Physical activity behaviours (activity level: activity counts per minute; activity variability: highest activity counts per minute each day divided by activity counts per minute on that day) were measured with accelerometers. Self-reported activity pacing (Activity Pacing and Risk of Overactivity Questionnaire), fatigue severity (Fatigue Severity Scale) and health-related quality of life (RAND-12-Item Short-Form Health Survey) were measured. Scatter plots were used to explore associations between measures.

**Results:**

Activity level was 258 ± 133 counts per minutes, activity variability was 4 ± 1, self-reported activity pacing was 3 ± 1, fatigue severity was 5 ± 2 and health-related quality of life was 43 ± 8. Increased self-reported activity pacing was associated with lower activity levels and less variability in daily activities.

**Conclusion:**

This investigation suggests that people with multiple sclerosis who have low physical activity levels could be inappropriately using activity pacing as a reactionary response to their multiple sclerosis symptoms.

## Introduction

Regular participation in physical activity is considered a central component of a healthy lifestyle for people with multiple sclerosis, being associated with improvements in multiple sclerosis fatigue symptoms, quality of life and maintenance of physical function.^1–5^ However, the experience and expectations of fatigue sensations in relation to physical activity can be a disabling symptom in people with multiple sclerosis and draws several activity engagement strategies.^6–9^ These include reduced activity levels resulting from and in anticipation of fatigue, and engaging in too many or prolong periods of activities when feeling better, resulting in worsening of fatigue symptoms and then needing to rest or be inactive for prolonged periods to recover.^6–9^ Therefore, to engage people with multiple sclerosis in an active lifestyle, it is crucial to explore strategies for managing fatigue symptoms, to lessen its adverse impact on physical activity behaviours.

Activity pacing (defined as breaking up one’s daily activities into more manageable portions, in a way that should not exacerbate symptoms, which then allows gradual progressive increases in activity) can help in management of symptoms and improve physical activity in people with multiple sclerosis.^7–13^ However, little is known about how the pacing strategies that people with multiple sclerosis enact in daily life influence their physical activity behaviour. The few available studies on activity pacing are inconclusive.^6–9^ While some studies show that activity pacing is associated with worse symptoms and disability,^8,9^ others found opposite or no associations.^11–14^ Studies to further the understanding of activity pacing are relevant to the development of behavioural interventions for people with multiple sclerosis to manage their fatigue symptoms and improve their participation in physical activity. The aim of this study was to explore the association between objectively-measured physical activity behaviours (including activity variability, i.e. fluctuation in daily activity level) and self-reported engagement in activity pacing in adults with multiple sclerosis. We hypothesised that self-reported engagement in activity pacing would be associated with higher activity levels and less variability in daily activities.

## Method

The study was approved by the University of Essex Ethics Committee, reference number 17/BS/499/AU. Data collection took place between July 2017 and December 2017. University of Essex was responsible for the integrity and conduct of the study, which had no funding.

Participants were recruited locally from Multiple Sclerosis-UK and Multiple Sclerosis Society in Colchester through public advertisements. Interested participants were contacted by the researchers who explained the study rationale, potential benefits, procedures and answered all questions. Criteria for inclusion were: people were 18 years and older, diagnosed with multiple sclerosis, been relapse-free during the last 30 days, not currently or recently (in the previous 12 months) engaged in a physical activity programme with or without activity management instruction, ambulatory (with or without assistive device) and English-speaking. Participants were excluded from the study if they were not able to complete the questionnaires, even with help, or had comorbid conditions with potential to influence daily physical activity levels. Eligible participants signed an informed consent form.

Enrolled participants were assessed through standardised baseline measurements obtained from two clinic visits. During the first visit, demographic data were collected. These included age, sex, body mass index calculated from self-reported body mass and height ((body mass (kg)/height^2^ (m^2^)), multiple sclerosis type (i.e. relapsing remitting, secondary progressive or primary progressive), duration of illness (years since diagnosis) and physical disability, assessed using the Patient Determined Disease Steps (PDDS).^15^ The PDDS is a valid patient-reported outcome of disability in multiple sclerosis and is strongly correlated with the Expanded Disability Status Scale.^16^ Participants then wore an accelerometer for seven days during a home monitoring period. Participants were instructed to wear the accelerometer at all times except on occasions when it could become wet (e.g. showering or swimming). After the home monitoring period, participants returned the accelerometer and completed a set of questionnaires: a self-report questionnaire on their activity pacing^12,17^ and short questionnaires on fatigue^18^ and health-related quality of life.^19^

Physical activity behaviour was assessed with waist-worn triaxial accelerometers (ActiGraph GT3X+, LLC, Fort Walton Beach, FL).^20,21^ Physical activity level was calculated by averaging the cumulative activity counts per minute over seven days. Activity variability which indicates the fluctuating nature of the physical activity pattern throughout the day,^22^ was calculated as the amount of physical activity during the peak activity hour for each day (identified as the hour with the highest number of activity counts), divided by the mean amount of physical activity on that day, and averaged over seven days. A high activity variability indicates a stronger concentration of physical activity each day, while a low activity variability suggests spread of physical activity more evenly throughout the day.

Self-reported engagement in activity pacing was evaluated with the self-reported engagement in activity pacing sub-scale of the Activity Pacing and Risk of Overactivity Questionnaire (Appendix A).^12,17^ Further details of the questionnaire are described elsewhere in literature.^12^ Fatigue severity was measured using the Fatigue Severity Scale,^18^ a reliable and valid measurement of the impact of fatigue in people with multiple sclerosis.^18,23,24^ Health-related quality of life was assessed by the RAND-12-Item Short-Form Health Survey Questionnaire,^19,25^ using the recommended scoring algorithm for estimating global health.^25–27^

All statistical analyses were performed using version 25.0 of the IBM Statistical Package for the Social Sciences (SPSS) software.^28^ All values are reported using descriptive statistics of means ± standard deviation to summarise characteristics of participants. Shapiro–Wilk test and visually inspecting Q-Q plots showed data were generally normally distributed. Where they were not, the median and interquartile range is presented. Associations between self-reported engagement in activity pacing and physical activity behaviours were examined using scatter plots.

## Results

In total 21 participants took part in the study. Sample characteristics and outcome date are displayed in Table 1. The sample reported clinically significant levels of fatigue severity (fatigue severity score >4)^29^ and moderate disability. Mean body mass index indicated that the sample was, on average, slightly overweight according the World Health Organization standards (Body mass index ⩾25.00).

**Table 1.** Demographics of participants.

Variable		Range
Number of participants	21	
Age, years (*M* ± SD)	59.33 ± 8.67	41.00–71.00
Body mass index, kg/m^2^ (median, IQR)	25.20 (3.40)	21.50–35.90
Sex, number of men (%)	15 (71.42)	
Multiple sclerosis type, number of RRMS (%)	11 (52.38)	
Number of PPMS (%)	9 (42.86)	
Number of SPMS (%)	1 (4.76)	
Disease duration, year (*M* ± SD)	14.57 ± 11.84	1–38.00
Patient determined disease step (*M* ± SD)	3.10 ± 1.26	1–6
Health-related quality of life^a^ (*M* ± SD)	42.66 ± 8.13	31.17–57.07
Engagement in pacing^b^ (*M* ± SD)	3.25 ± .74	1.60–4.60
Perceived risk of overactivty^b^ (*M* ± SD)	3.38 ± 1.02	1.00–5.00
Fatigue severity^c^ (*M* ± SD)	4.75 ± 1.62	1.00–7.00
Physical activity counts^d^ (*M* ± SD)	257.97 ± 131.58	71.86–636.33
Physical activity variability^d^ (*M* ± SD)	3.96 ± .72	2.87–5.93

M: mean; PPMS: primary progressive multiple sclerosis; SPMS: secondary progressive multiple sclerosis; RRMS: relapsing remitting multiple sclerosis; SD: standard deviation; IQR: interquartile range.

a RAND-12 Health Survey.

b Activity Pacing and Risk of Overactivity Questionnaire.

c Fatigue Severity Scale.

d ActiGraph accelerometer.

**Figure 1.**
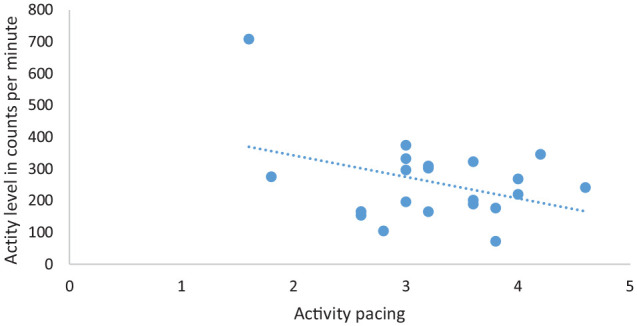
Scatter plot of self-reported engagement in activity pacing and physical activity levels of study participants (*N* = 21).

**Figure 2.**
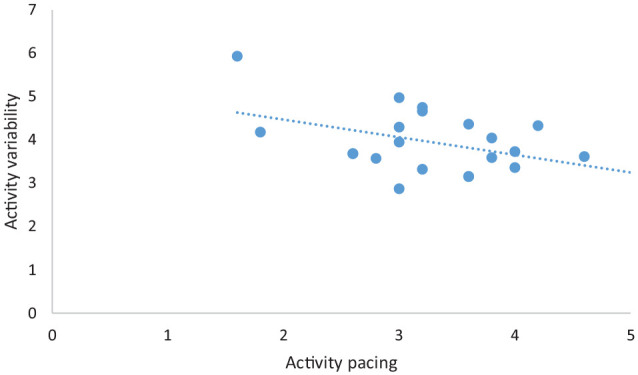
Scatter plot of self-reported engagement in activity pacing and physical activity variability (fluctuation in physical activity levels) of study participants (*N* = 21).

A visual inspection of the scatter plots of self-reported engagement in activity pacing and activity levels (Figure 1), and self-reported engagement in activity pacing and activity variability (Figure 2) revealed reported use of activity pacing was associated with lower activity levels and lower activity variability. In other words, people who engage more in activity pacing had lower activity levels and lower activity variability.

## Discussion

This study investigated the associations between self-reported engagement in activity pacing and physical activity levels and physical activity variability (fluctuation in daily activity level) to further our understanding of activity pacing, and found weak negative associations between self-reported engagement in activity pacing and physical activity levels, and between self-reported engagement in activity pacing and activity variability. In other words, increased use of self-reported engagement of activity pacing was associated with lower physical activity levels and lower activity variability. The finding that the use of activity pacing was associated with lower physical activity levels was similar to the findings of studies investigating whether there was a relationship between the use of activity pacing and physical activity within daily routines in persons with osteoarthritis.^8,14^ The finding that increased use of activity pacing was associated with less daily variability in activity was contrary to that reported by Murphy et al.^30^ in their study of the association between activity pacing and activity variability in a sample of adults with hip or knee osteoarthritis. In their study, the authors reported no association between activity pacing and activity variability.

Descriptive statistics showed the study sample reported, clinically significant fatigue severity scores, which was similar to studies evaluating fatigue severity in the MS population, high use of engagement in activity pacing and a high perceived risk of preventing overactivity.^12,13,29^ The physical activity counts per minutes reported by our study sample is consistent with previous research involving people with MS.^20,31^ The fatigue severity score (4.75 ± 1.62) reported by our study sample was comparable with those reported in other studies involving people with MS.^12,13,23,32^ In their study, Merkelbach et al.^23^ reported a mean FSS score of 4.4 ± 1.6. The clinically significant fatigue severity reported by our sample coupled with our finding that increased self-reported use of engagement in activity pacing was associated with lower activity levels suggests that people with multiple sclerosis who experience more disruption through fatigue symptoms in daily life may either be using activity pacing as reactionary response to limiting their activity due to increased fatigue sensation or in anticipation to imminent increase in fatigue sensation. This is consistent with the notion that avoiding or limiting activity may be a reactionary response to increased symptoms and associated with lower physical activity levels.^8,30^

People with multiple sclerosis who experience more disruption through fatigue in daily life may be consciously limiting their activities to prevent flares in fatigue symptoms. Conversely, people with multiple sclerosis who experience less disruption through fatigue symptoms in daily life might be prone to engaging in too many or prolonged periods of activities and then experience the adverse consequences of overactivity. With both underactivity and overactivity, and subsequent increased variability in daily activity associated with disability,^9,30^ people with multiple sclerosis need to avoid both over-exertion and under-exertion. Activity pacing is recommended as a plausible strategy to prevent over-exertion and under-exertion.^12,13^ As activity pacing is aimed to maintain a steady optimal activity level and reduce periods of high activity that could lead to a flare in fatigue symptoms, it is expected that people who pace their activities would have less variability in their daily activity.^30^ Consequently, there seems to be a need for guidance on the use of activity pacing as a means to maintain optimal activity levels and avoid the deconditioning effects of a multiple sclerosis diagnosis^32^ and to improve multiple sclerosis symptoms such as fatigue, rather than engaging in avoidance behaviour to manage symptoms. It is notable that increased self-reported use of activity pacing was associated with less variability in daily activity.

Further research to understand the differential needs of subtypes of people with multiple sclerosis, to help the development of behavioural interventions aimed at building the skills and confidence needed to effectively manage daily physical activity levels, thereby optimising the health benefits in relation to multiple sclerosis symptoms is needed. This study had a number of limitations. Importantly, because this sample population was recruited from a single catchment area of the UK, the findings are limited in their generalizability to a more diverse multiple sclerosis population. In addition, the small sample size, atypical high percentage of men and older people with multiple sclerosis are additional study limitations, as multiple sclerosis affects almost three times as many women as men and most people are diagnosed between the ages 20 and 40 years. The main strength of the study is the novel approach used to explore the association between self-reported free-living activity pacing during daily life and objectively-measured physical activity behaviour.

## Conclusion

In this study, we investigated the associations between physical activity behaviour (using accelerometry) and self-reported engagement in activity pacing in the daily routines of adults with multiple sclerosis. We found lower physical activity levels and activity variability were associated with increased self-reported engagement in activity pacing. The results of the study yielded a preliminary insight into activity pacing in relation to physical activity and fatigue perception amongst people with multiple sclerosis, which provides a platform for further research into tailored physical activity interventions incorporating fatigue management. Such interventions would be aimed at re-educating people with multiple sclerosis on how activity pacing could be used to increase physical activity levels as a means of improving symptoms, rather than using activity pacing as a physical activity avoidance strategy to manage symptoms.

**Clinical messages**

- An increase in self-reported engagement in activity pacing was associated with lower physical activity levels and less variability in people with multiple sclerosis experiencing clinically significant fatigue severity.
- People with multiple sclerosis who experience more disruption through fatigue symptoms in daily life seem to naturally use activity pacing to limit their activities in response to increased fatigue sensations or anticipation of imminent increase in fatigue sensation.

## Appendix A

**Table A1.** Factor loadings of the seven items of the Activity Pacing and Risk of Overactivity Questionnaire using Principal Component Analysis with oblique rotation.

Items	Factor 1	Factor 2
A. During the day I plan several moments to recover.	0.73*	0.04
B. I perform my activities at a slow pace.	0.65*	−0.13
C. When performing my activities, I take my fatigue into account.	0.79*	0.00
D. When I’m engaged in an activity, I find it difficult to stop timely.	0.05	0.88*
E. I alternate intensive activities with less intensive activities.	0.70*	0.08
F. I divide my activities over the day.	0.74*	−0.05
H. I find it hard to limit my activities.	−0.06	0.87*

Factor 1: Engagement in pacing.

Factor 2: Perceived risk of overactivity.

* Loadings that can be explicitly assigned to a single factor (factor loading >0.40).
